# Novel multivalent design of a monoclonal antibody improves binding strength to soluble aggregates of amyloid beta

**DOI:** 10.1186/s40035-021-00258-x

**Published:** 2021-09-28

**Authors:** Fadi Rofo, Jos Buijs, Ronny Falk, Ken Honek, Lars Lannfelt, Anna M. Lilja, Nicole G. Metzendorf, Tobias Gustavsson, Dag Sehlin, Linda Söderberg, Greta Hultqvist

**Affiliations:** 1 Protein Drug Design, Faculty of Pharmacy, Uppsala University, 75124 Uppsala, Sweden; 2 Department of Immunology, Genetics and Pathology, Uppsala University, 75185 Uppsala, Sweden; 3 Ridgeview Instruments, 75237 Uppsala, Sweden; 4 BioArctic AB, 11251 Stockholm, Sweden; 5 Department of Public Health and Caring Sciences, Uppsala University, 75185 Uppsala, Sweden

**Keywords:** Multivalent antibodies, Alzheimer’s disease, Aβ, Avidity, Oligomers, Protofibrils

## Abstract

**Background:**

Amyloid-β (Aβ) immunotherapy is a promising therapeutic strategy in the fight against Alzheimer’s disease (AD). A number of monoclonal antibodies have entered clinical trials for AD. Some of them have failed due to the lack of efficacy or side-effects, two antibodies are currently in phase 3, and one has been approved by FDA. The soluble intermediate aggregated species of Aβ, termed oligomers and protofibrils, are believed to be key pathogenic forms, responsible for synaptic and neuronal degeneration in AD. Therefore, antibodies that can strongly and selectively bind to these soluble intermediate aggregates are of great diagnostic and therapeutic interest.

**Methods:**

We designed and recombinantly produced a hexavalent antibody based on mAb158, an Aβ protofibril-selective antibody. The humanized version of mAb158, lecanemab (BAN2401), is currently in phase 3 clinical trials for the treatment of AD. The new designs involved recombinantly fusing single-chain fragment variables to the N-terminal ends of mAb158 antibody. Real-time interaction analysis with LigandTracer and surface plasmon resonance were used to evaluate the kinetic binding properties of the generated antibodies to Aβ protofibrils. Different ELISA setups were applied to demonstrate the binding strength of the hexavalent antibody to Aβ aggregates of different sizes. Finally, the ability of the antibodies to protect cells from Aβ-induced effects was evaluated by MTT assay.

**Results:**

Using real-time interaction analysis with LigandTracer, the hexavalent design promoted a 40-times enhanced binding with avidity to protofibrils, and most of the added binding strength was attributed to the reduced rate of dissociation. Furthermore, ELISA experiments demonstrated that the hexavalent design also had strong binding to small oligomers, while retaining weak and intermediate binding to monomers and insoluble fibrils. The hexavalent antibody also reduced cell death induced by a mixture of soluble Aβ aggregates.

**Conclusion:**

We provide a new antibody design with increased valency to promote binding avidity to an enhanced range of sizes of Aβ aggregates. This approach should be general and work for any aggregated protein or repetitive target.

**Supplementary Information:**

The online version contains supplementary material available at 10.1186/s40035-021-00258-x.

## Background

Immunotherapy is one of the fastest growing fields in medical research. The success in drug development is partly due to the capability of antibodies to bind their protein targets with high specificity and affinity. Since IgG antibodies have two identical paratopes for the same epitope, they can bind repetitive targets (e.g. oligomeric Aβ) at multiple sites (epitopes). As the number of connected binding sites on one target increases, the rate of dissociation from the target will decrease. This effect is called avidity, which is defined as the unified strength of multiple interactions between an antibody and its target [1, 2]. The more engaged binding sites, the greater the avidity. For example, IgM antibodies with 10 binding sites can have higher avidity than IgG antibodies, when multiple epitopes on a target are bound simultaneously. It has been shown that antibodies modified into a tetravalent form have higher avidity than bivalent antibodies [3].

Protein aggregation is one of the major pathological hallmarks of several diseases. In Alzheimer’s disease (AD), the most common form of neurodegenerative disorders, amyloid beta (Aβ) forms aggregates, which cause neuronal death and lead to impaired memory [4]. Aggregation of the monomeric Aβ proceeds through the formation of intermediate soluble oligomers and protofibrils, and eventually lead to formation of insoluble fibrils and plaques, mainly located in the extracellular space in the brain [5–8]. The latter are easily visible in the post-mortem brains from AD patients when labeled with amyloid-binding dyes [9]. However, several studies have shown that the soluble aggregated species (oligomers and protofibrils) are the most toxic forms, since they correlate better with clinical symptoms and have been shown to be the main species responsible for the associated neuronal and synaptic death [7, 10–12].

Passive immunization with anti-Aβ antibodies as a treatment strategy for AD has been tested in numerous clinical trials [13, 14]. One of the main challenges with AD immunotherapy is to create an antibody with strong binding to toxic species of Aβ, and less binding to the potentially physiologically relevant monomers or insoluble fibrils. The monomers may exert some neuroprotective effects [15, 16]. It is debated which aggregated species of Aβ are most harmful. Some studies suggest soluble protofibrils [17], while others suggest smaller oligomers [10, 18] or dimers [19, 20]. A general conclusion is that soluble Aβ aggregates are toxic to the cells and hence a relevant target. Smaller oligomers of Aβ are more likely to enter neurons and thereby exert their neurotoxic effects, while larger aggregates like protofibrils might have more indirect toxic effects by for example enhancing neuroinflammation [21].

The binding-selectivity of anti-Aβ antibodies to aggregated species of Aβ over monomers is mainly dependent on avidity. Several antibodies can strongly bind to Aβ aggregates while having a low affinity to monomers via the avidity effect. Examples of such binders include IgM antibodies with 10 binding sites [22, 23], divalent binders [24] and antibodies reaching phase 3 clinical trials like aducanumab [25] and lecanemab (BAN2401) [26, 27]. Increasing the valency of antibodies could potentially increase the interaction time with the targeted antigen if the additional binding sites are able to bind to the target simultaneously. If one paratope is dissociated, there will still be a second paratope in association with the target, which could also increase the chance of the dissociated paratope to bind again to the target [2]. Efforts have been made to increase the valency of antibodies through designing multimeric formats accompanied with a significant decrease in the dissociation rate constants [28, 29]. However, despite being highly flexible in structure [30], IgG antibodies may not have an avidity effect to small Aβ oligomers [31] due to the spatial distance between the two paratopes of IgG antibodies, which has been previously determined to be around 100 Å [32]. As illustrated in Additional file 1: Fig. S1, the  complementarity-determining regions of an IgG4 antibody (PDB ID 5DK3) in its crystal structure are much larger than a 12-mer Aβ (PDB ID 2BEG). However, different subclasses of IgG antibodies have different degrees of flexibility, which should be considered when looking at the interactions of specific antibodies with Aβ [33].

In this study, we set out to develop new functional multivalent antibodies to enhance antibody avidity to soluble aggregates of Aβ, particularly smaller oligomers, while maintaining a weak binding to monomers. For this reason, we have designed and recombinantly produced antibody formats based on an Aβ protofibril antibody [26, 27]. This antibody has strong binding to Aβ protofibrils, moderate binding to insoluble fibrils and weak binding to monomers [34–36]. In this study, we introduced new designs involving engineering additional binding domains (e.g. single-chain fragment variable [scFv]) on the variable domains (binding sites) of the parental antibody, generating tetra- and hexavalent antibodies, and tested their binding to different species of Aβ aggregates and protective effects on cells.

## Materials and methods

### Antibody cloning, expression and purification

The heavy and light chains of the different antibodies were cloned into two separate pcDNA3.4 vectors (GeneArt, Regensburg, Germany). The recombinant proteins were expressed as described previously [37]. Briefly, Expi293 cells were transfected with 70% light chain plasmids and 30% heavy chain plasmids using polyethyleneimine as a transfection agent. Seven to 12 days after transfection, the antibodies were purified using an Äkta start system with protein G columns (Cytiva, Uppsala, Sweden). Acetic acid at 0.7% was used as an elution buffer, and absorbance at 280 nm was used to measure the concentrations of purified antibodies. Buffer was exchanged to phosphate-buffered saline (PBS) using Zeba desalting columns (Pierce biotech, Rockford, IL) and the antibodies were stored  at -80 °C until further application.

### SDS-PAGE analysis

SDS-PAGE analysis was performed to confirm the purity and the size of the purified proteins. The antibodies were mixed with LDS sample buffer (Invitrogen, Waltham, MA) and loaded onto 4%–12% Bis–Tris protein gels (Invitrogen, Waltham, MA) without adding reducing agents. The gel was then stained with PAGE blue protein solution (Thermo Scientific, Waltham, MA) using a pre-stained protein marker (LI-COR biosciences, Bad Homburg, Germany) as a molecular weight standard.

### Thermal shift assay

The structural stability of the generated antibodies under thermal stress was evaluated using Tycho nt.6 instrument (NanoTemper Technologies, München, Germany). Equimolar concentrations of the antibodies were heated in a glass capillary where the temperature increased linearly from 35 °C to 95 °C. Fluorescence intensities at 330 nm and 350 nm were recorded, corresponding to tryptophan fluorescence. The assay was performed under three conditions: immediately after thawing the antibodies, following  2-h incubation at 37 °C and following  72-h incubation at 37 °C.

### Labelling of the antibodies with Iodine-125

The recombinant antibodies were labelled with iodine-125 using Chloramine-T as described previously [38]. Briefly, equal amounts of RmAb158 (molecular weight [MW] 150 kDa), Tetra-RmAb158 (MW 200 kDa) and Hexa-RmAb158 (MW 250 kDa) were mixed with the iodine-125 stock solution (Perkin Elmer Inc, Waltham, MA) and 1 mg/ml of Chloramine-T (Sigma Aldrich, Stockholm, Sweden) in PBS. After 90 s, the reaction was stopped by addition of 1 mg/ml sodium meta-bisulphite (Sigma Aldrich, Stockholm, Sweden). Free and unbound iodine was removed from the labelled antibodies using Zeba mini desalting columns having a MW cut-off of 7 kDa (Pierce biotech, Rockford, IL) and the antibodies were eluted in PBS.

### Real-time interaction analysis with LigandTracer

The binding properties of the antibodies to protofibrils were studied using LigandTracer Grey (Ridgeview Instruments AB, Uppsala, Sweden). Briefly, a Petri dish was coated with protofibrils in a defined (target) area and placed on a tilted, rotating support. The area opposite to the target area was defined as the background area. The LigandTracer Grey has a low-energy gamma detector mounted above the upper part of the dish. When the buffer containing radio-labeled antibodies was added to the dish, the inclination ensures that the liquid was mainly in the lower part of the dish outside the detection area. During each full rotation, the decay corrected signals from the target and the background areas were recorded for 30 s each. Each recording was delayed for 5 s to allow the buffer to drain from the area being detected. The background signal was subtracted from the target signal to represent specific binding of labeled antibodies to protofibrils. To this end, Petri-dishes (Corning Inc., Corning, NY) were coated overnight at 4 °C with 300 μl of 500 nM Aβ1-42 protofibrils, prepared as described previously [31]. The dishes were blocked with 5% BSA in 1 × PBS for 2 h at room temperature (RT). To establish the affinity and kinetics of the interaction processes, protofibrils were incubated with two antibody concentrations (0.3 and 1 nM in 2 ml of 0.1% BSA/PBS) for 2.5 h and 3 h, respectively. After a total incubation of 5.5 h, the dissociation measurement was initiated by removing the antibody-containing solution and adding 3 ml of 0.1% BSA/PBS buffer to the dish. Data evaluation was performed using TraceDrawer software (Ridgeview Instruments AB, Uppsala, Sweden). The labelling procedure was performed roughly two hours before starting the first incubation.

### Surface plasmon resonance (SPR)

The binding strength of the recombinant antibodies to Aβ protofibrils and monomers was further investigated by SPR using Biacore 8 K instrument (Cytiva, Uppsala, Sweden). Soluble Aβ1-42 protofibrils, prepared as described previously [31], were immobilized by amine coupling (NHS/EDC, GE kit #BR100633) on a biacore CM5 sensor chip (Cytiva, Uppsala, Sweden). Three-fold dilution series of the recombinant antibodies (ranging from 100 to 1.23 nM) in  PBS-P+ buffer (pH 7.4) (Cytiva, Uppsala, Sweden) were injected using a single cycle kinetic method at a flow rate of 30 μl/min with an association phase of 120 s, followed by a dissociation phase of 7200 s. Injection of buffer was used as a blank control. The binding data were fitted to a 1:1 kinetic model using Biacore Insight Evaluation. The dissociation rate constant was calculated as an average value from 2–5 measurements. Surfaces were regenerated using 3 M MgCl_2_ between cycles. For binding to monomers, the recombinant antibodies were immobilized on the mentioned chips at a fixed concentration of 1 μg/ml. A single cycle kinetic method was used to inject a two-fold dilution series of Aβ1-40 monomers (ranging from 4000 to 250 nM) (Bachem, H-1194, Bubendorf, Switzerland). The steady-state kinetic model was used for analysis.

### Indirect ELISA to demonstrate the avidity of RmAb158 to Aβ protofibrils

Ninety-six-well half-area plates (Corning Inc., Corning, NY) were coated overnight with Aβ1-42 protofibrils (45 ng/well), prepared as described previously [31], at 4 °C. The plates were blocked the next day with 1% BSA in PBS for 2 h at RT, followed by incubation for an additional 2 h at RT with serial dilutions of RmAb158 (with a starting concentration of 10 nM) and Fab fragment of RmAb158 (with a starting concentration of 1.25 μM). A polyclonal horse-radish peroxidase (HRP)-conjugated anti-mouse IgG (Sigma-Aldrich, Stockholm, Sweden) was added to the plates and incubated for 1 h at RT, followed by signal development with K-blue aqueous TMB (Neogen Corp, Lexington, KY). Absorbance was measured at 450 nm using a FLUOstar Omega microplate reader (BMG Labtech, Ortenberg, Germany).

### Generation of cross-linked stabilized Aβ1-42 aggregates and separation through size exclusion chromatography (SEC)

Synthetic Aβ1-42 (Bachem, Bubendorf, Switzerland) was dissolved in 10 mM NaOH supplemented with 0.005% Tween-20 to a final concentration of 100 µM and stored in aliquots at − 80 °C. For generation of Aβ1-42 aggregates, the stock dilution was diluted in 2 × PBS, pH 7.4, to a final concentration of 50 µM. The 50 µM solution was incubated without shaking for 15 min at 37 °C to generate Aβ1-42 aggregates. For stabilization of the metastable Aβ1-42 aggregates, the aggregates were stabilized covalently by the Photo-Induced Cross-linking of Unmodified Proteins (PICUP) method according to a protocol described previously [39]. Mechanistically, PICUP involves photo-oxidation of Ru3 + in a tris(bipyridyl)Ru (II) complex (RuBpy) to Ru3 + by irradiation with visible light in the presence of an electron acceptor. Briefly, a typical PICUP reaction was performed in a 50 µl reaction volume. To the 50 µM Aβ1-42 aggregate solution, 5 µl of RuBpy (2.5 mM dissolved in water) followed by 5 µl of ammonium persulfate (APS, 10% (*w*/*v*) in water) was added by pipetting (final concentrations of RuBpy and APS in the reaction mixture were 0.25 mM and 1%, respectively). The solution was quickly irradiated for 5 s under a general light bulb on the lab bench. Immediately after irradiation, the reaction was quenched by separating the reaction mixture with Zeba spin desalting column 7 k MWCO equilibrated with PBS supplemented with 0.005% Tween-20 (Thermofisher, Waltham, MA). The PICUP stabilized Aβ1-42 aggregates were heat-treated for 5 min at 95 °C prior to separation of Aβ1-42 aggregates by SEC. A Superdex 200 Increase 3.2/300 column (Cytiva, Uppsala, Sweden) was used for size separation of the first batch of Aβ1-42 aggregates (Fig. 6) on a Merck Hitachi D-700 HPLC LaChrom system. The second batch of Aβ1-42 aggregates (Additional file 1: Fig. S3a) was separated using a Superdex 75 column (Cytiva, Uppsala, Sweden). The samples were eluted with PBS-Tween, pH 7.4 (50 mM sodium phosphate, 0.15 M NaCl, 0.1% Tween-20, pH 7.4) at a flow rate of 0.08 ml/min and data obtained at 214 nm. Before sample injection, the quenched reaction mixture was mixed 1:1 with a 2 × mobile-phase buffer (100 mM sodium phosphate, 0.3 M NaCl, 0.2% Tween-20, pH 7.4). Fractions were collected every two minutes and stored at − 20 °C for further analysis. To estimate the size of the separated PICUP Aβ1-42 fractions, western blot analysis was performed. Samples were mixed with Laemlli sample buffer containing reducing agent and heated for 5 min at 95 °C. The denaturated samples were loaded on NuPAGE 12% Bis–Tris gels (Thermofisher, Waltham, MA), and transferred onto nitrocellulose membranes (Bio-Rad, Hercules, CA). The membranes were blocked with 5% dry milk (Bio-Rad, Hercules, CA) in TBS-Tween buffer, followed by incubation with rabbit anti-Aβ42 antibody (Bioarctic AB, Stockholm, Sweden).

### Sandwich ELISA to check the binding strength to cross-linked Aβ1-42 aggregates and α-synuclein aggregates

The generated antibodies were tested with sandwich ELISA to establish their binding strength to different sizes of the generated cross-linked Aβ1-42 aggregates using the N-terminal specific mouse monoclonal antibody 82E1 (IBL/Tecan Trading AG, Mannedorf, Switzerland) as a positive control. Ninety-six-well half-area plates (Corning Inc., Corning, NY) were coated overnight with 1 μg/ml of the rabbit polyclonal C-terminal-specific Aβ1-42 antibody (Invitrogen, Waltham, MA) at 4 °C. On the next day, the plates were blocked with 1% BSA in PBS for 2 h at RT and incubated with 250 pM of the generated cross-linked Aβ1-42 aggregates of different sizes for another 2 h. Serial dilutions of the recombinant antibodies were added to the plates and incubated for 2 h at RT, followed by detection for 1 h at RT with HRP-conjugated anti-mouse IgG antibody (Sigma Aldrich, Stockholm, Sweden). Signals were developed with K-blue aqueous TMB (Neogen Corp, Lexington, KY) and the absorbance was measured at 450 nm using a FLUOstar Omega microplate reader (BMG Labtech, Ortenberg, Germany). The wells were washed with ELISA washing buffer (PBS with 0.05% Tween-20) between each step. All serial dilutions were made with ELISA incubation buffer (PBS with 0.1% BSA and 0.05% Tween-20). The absorbance of the blank (no addition of recombinant antibodies) was subtracted from the absorbance of the samples. For α-synuclein sandwich ELISA, plates were coated overnight with 1 μg/ml of antibody MJFR-14–6-4–2 (Abcam, Cambridge, United Kingdom) at 4 °C. Following block with 1% BSA in PBS for 2 h at RT, α-synuclein oligomers, prepared as described previously [40], were added at a final concentration of 10 nM and the plates were further incubated for 2 h at RT. Serial dilutions of the recombinant antibodies (RmAb158, Tetra-RmAb158 and Hexa-RmAb158) were added to the plate and incubated for 2 h at RT. The α-synuclein antibody (clone SynO2, recombinantly produced in the lab) was used as a positive control [41]. The signals were developed and detected as described above.

### Inhibition ELISA to discriminate the binding between Aβ monomers, oligomers, protofibrils and fibrils

Inhibition ELISA was performed as described previously [34]. Briefly, 96-well half-area plates (Corning Inc., Corning, NY) were coated overnight with 45 ng/well of Aβ1-42 protofibrils at 4 °C followed by blocking with 1% BSA in PBS for 2 h at RT. Serial dilutions of sonicated Aβ1-42 insoluble fibrils, prepared as described previously [31], large cross-linked Aβ1-42 protofibrils (fraction 1 in SEC chromatogram, Fig. 6), cross-linked Aβ1-42 oligomers (fractions 3 and 4 in SEC chromatogram, Fig. 6) and Aβ1-40 monomers (Bachem, Bubendorf, Switzerland) were pre-incubated in a non-binding 96-well plate with HexaRmAb158 at a fixed concentration of 250 pM. After 1.5-h incubation, the mixtures were transferred to the Aβ protofibril-coated plates and further incubated for 15 min at RT. The plates were washed between the steps and the signal was developed and detected as described above.

### Cell culture and MTT assay

The mouse neuroblastoma Neuro2a cell line was obtained from ATCC (Manassas, VA) and grown in Minimum Essential Medium (Gibco, Carlsbad, CA) supplemented with 10% fetal bovine serum (Gibco, Carlsbad, CA) at 37 °C with 5% CO_2_. The cells were plated in a 96-well plate (Sarstedt cells + , Numbrecht, Germany) at a density of 5000 cells in 100 µl complete medium per well. To eliminate the effect of serum, the cells were starved 24 h prior to the addition of Aβ/antibody mixtures by changing to a serum-free medium. Cells were then treated with a heterogenous mixture of cross-linked Aβ1-42 aggregates (the sample before being passed through SEC, final concentration 500 nM) either alone or in combination with the antibodies (final concentration 150 nM). Cells incubated at standard conditions for 24 h with PBS were used as a negative control and with 0.005% H_2_O_2_ as a positive control. After the mentioned incubation time, the treatment medium was discarded and 50 μl serum-free medium plus 50 μl MTT reagent (Abcam, Cambridge, United Kingdom) was added in each well and incubated for 3 h at standard conditions, which lead to the formation of formazin proportional to how many cells that are alive. To solubilize the formazin product, MTT solvent (Abcam, Cambridge, United Kingdom) was added and the plates were incubated in the dark for 15 min while shaking. The absorbance was measured at 590 nm using a FLUOstar Omega microplate reader (BMG Labtech, Ortenberg, Germany). Data were obtained from two repetitive experiments where five to six replicates were used for each condition and the absorbance of the blank (cell medium only) was subtracted from the absorbance of the samples.

## Results

### Generation of recombinant multivalent antibodies

**Fig. 1:**
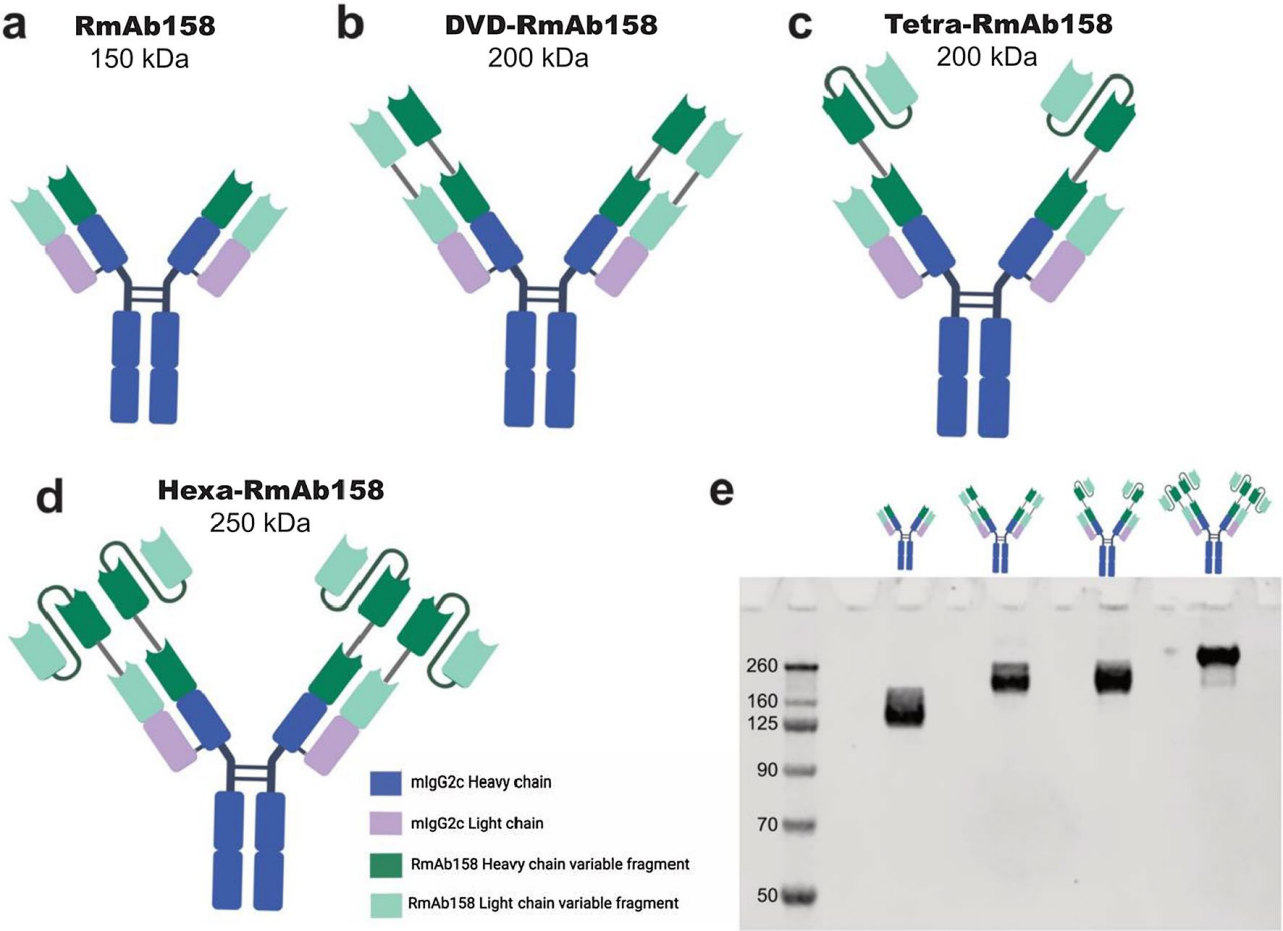
Design of the different recombinant antibodies. **a** Schematic picture of RmAb158 antibody. **b** DVD-RmAb158, where the heavy and light chain variable domains are added to the N-terminal end of Rmab158 heavy and light chains, respectively. **c** Tetra-RmAb158, where scFvs are added to the N-terminal end of Rmab158 heavy chain only. **d** Hexa-RmAb158, where scFvs are added to the N-terminal end of the heavy and light chains of RmAb158. **e** SDS-PAGE showing a band approximately at 150 kDa for RmAb158, 200 kDa for both DVD-RmAb158 and Tetra-RmAb158, and around 250 kDa for Hexa-RmAb158

A scFv, consisting of the heavy variable domain attached through a glycine-serine rich linker [42, 43] to the light variable domain of RmAb158 (Fig. 1a), was attached to the N-terminal end of the RmAb158 heavy chain to create Tetra-RmAb158 (Fig. 1c), or the N-terminal end of both heavy and light chains of RmAb158 to create Hexa-RmAb158 (Fig. 1d). The scFv was attached with an in-house designed linker where prolines were added to the linker to ensure no alpha helices were formed, glycines added to provide flexibility and serines added to give more hydrophilicity [44]. In addition, a dual variable domain (DVD) antibody was designed, where an additional heavy chain variable domain was attached to the N-terminal end of the heavy chain and an additional light chain variable domain was attached to the N-terminal end of the light chain (Fig. 1b).

The four antibodies were expressed in Expi293 cells [37], giving rise to ~ 20 mg of RmAb158 or 2–5 mg of DVD, Tetra or Hexa-RmAb158 per liter of transfected cell cultures. After purification with affinity column chromatography, SDS-PAGE analysis showed bands at 150 kDa (RmAb158), 200 kDa (DVD and Tetra-RmAb158) and 250 kDa (Hexa-RmAb158), confirming the size and purity of the produced recombinant proteins (Fig. 1e).

### Structural stability of the recombinant antibodies

**Fig. 2:**
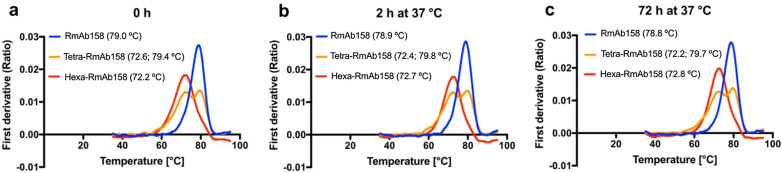
Structural stability of the recombinant antibodies. Stability of RmAb158, Tetra-RmAb158 and Hexa-RmAb158 under thermal stress evaluated with Tycho nt.6 system (NanoTemper Technologies, München, Germany). Inflection temperature(s) at which the proteins destabilize measured for the three antibodies (final concentration of 1 μM) at **a** 0 h, **b** 2 h, and **c** 72 h incubation at 37 °C. Results presented as the ratio of fluorescence intensity at 350 nm to 330 nm. Changes in fluorescence intensities reflect changes in the proteins’ structural stability. Due to the presence of scFv on their N-terminal ends, both Tetra-RmAb158 and Hexa-RmAb158 displayed lower inflection temperatures compared to RmAb158. Inflection temperatures of the three antibodies remained constant after 2 h and 72 h incubation at 37 °C, further suggesting structural stability of these antibodies. Results are generated from two-repetitive experiments and average values are shown

Structural stability of the generated antibodies under thermal stress was evaluated using the Tycho nt.6 system (NanoTemper Technologies, München, Germany). Inflection temperature which refers to the temperature at which the proteins unfold was determined by measuring fluorescence intensities at 350 and 330 nm. RmAb158 yielded an infection temperature of 79 °C (Fig. 2a). Two inflection temperatures were detected for Tetra-RmAb158 (72.6 °C and 79.4 °C) (Fig. 2a). Hexa-RmAb158 displayed a single inflection temperature of 72.2 °C (Fig. 2a). The slightly lower inflection temperatures detected for Tetra- and Hexa-RmAb158 antibodies can be attributed to the presence of scFvs on the N-terminal ends of these antibodies, as scFvs are more susceptible to thermal shifts than full IgG antibodies. The inflection temperatures of the three antibodies remained constant after incubation at 37 °C for 2 h (Fig. 2b) and 72 h (Fig. 2c), further suggesting the structural stability of the generated antibodies.

### RmAb158 selectivity for Aβ protofibrils is based on avidity

**Fig. 3:**
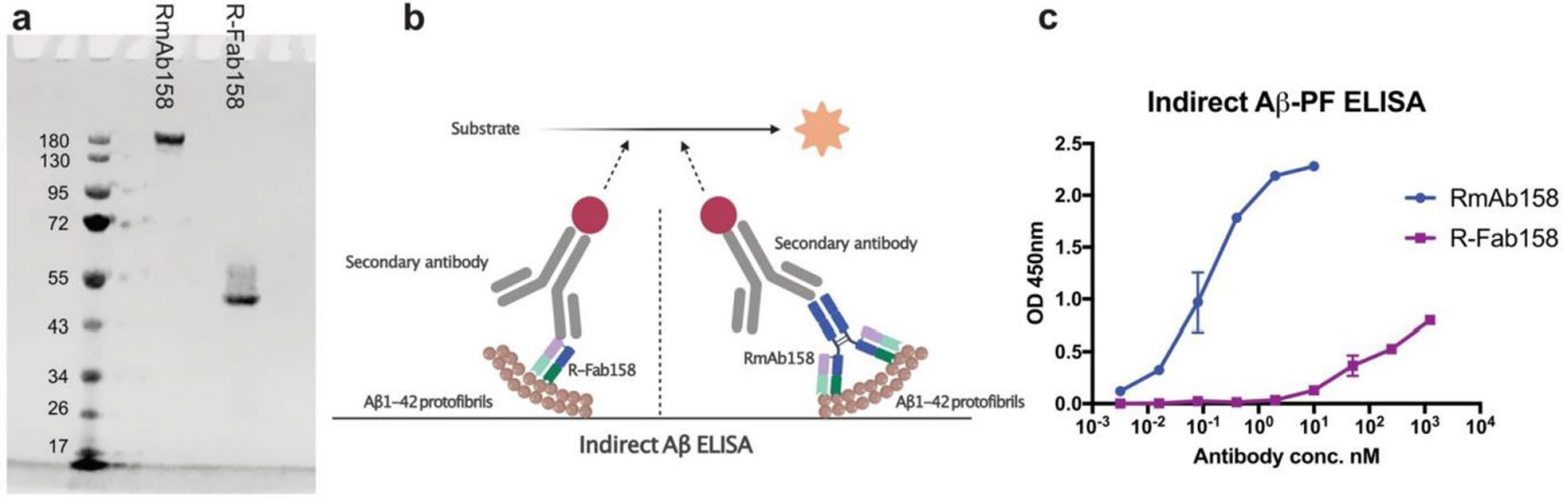
Studying the avidity of RmAb158 to Aβ protofibrils. **a** SDS-PAGE showing a single band approximately at 150 kDa for RmAb158 and 50 kDa for its Fab fragment, R-Fab158. **b** Schematic representation of indirect Aβ ELISA setup where Aβ1-42 protofibrils are coated on the well surface, followed by binding of the generated Rmab158 or R-Fab158. **c** Indirect ELISA displaying the binding curves of RmAb158 and R-Fab158 to Aβ1-42 protofibrils. Strong binding of RmAb158 to Aβ protofibrils compared to R-Fab158 that demonstrates weak binding. Data are presented as mean ± SD, *n* = 2

To demonstrate that RmAb158 utilizes the avidity effect to ensure strong binding to Aβ protofibrils but not monomers, the Fab fragments of the antibody were generated (Fig. 3a). Using an indirect ELISA setup (Fig. 3b), the binding strength to Aβ1-42 protofibrils was examined using the full RmAb158 antibody (MW 150 kDa), where both binding sites are available, and the Fab fragment of RmAb158 (MW 50 kDa), where only one binding site is available. A much stronger binding to Aβ protofibrils was observed for RmAb158 as compared to its Fab fragment alone (Fig. 3c). Hence, the strong binding that RmAb158 has to the protofibrils requires the binding of both arms to the same protofibril. This implicates the importance of avidity as a decisive factor in RmAb158’s binding selectivity to Aβ aggregates.

### Kinetic evaluation of binding of antibodies to Aβ1-42 protofibrils

**Fig. 4:**
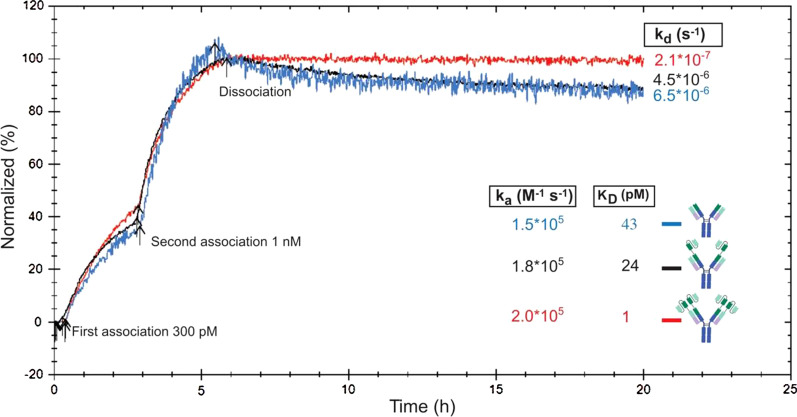
LigandTracer experiments illustrating the binding strength of the recombinant antibodies to Aβ1-42 protofibrils. Detection signal of iodine-125-labelled RmAb158 (blue), Tetra-RmAb158 (black) and Hexa-RmAb158 (red) to Aβ1-42 protofibrils, normalized to 100% corresponding to the maximum binding level, for evaluation of binding behavior and kinetics. Binding curves show association at 300 pM (antibody added at time point 0.5 h) and 1 nM (antibody added at time point 3 h), and dissociation (when the antibody was removed). Comparison of the affinity (*K*_D_), association rate constant (*k*_a_) and dissociation rate constant (*k*_d_) among the antibodies using one-to-one kinetic model. Results are generated from two repetitive experiments and average affinity constants are shown

The first question on our multivalent antibodies was if they could bind with high avidity to the rather large protofibrils (> 100 kDa in size). The affinity of RmAb158 to Aβ protofibrils has previously been estimated to be approximately 70 pM [35, 45], and the affinity of antibody formats with higher valency was expected to be even higher with a lower rate of dissociation. To verify this, the binding strength of the recombinant antibodies to Aβ1-42 protofibrils was determined with real-time interaction analysis using LigandTracer. For these experiments, iodine-125-labeled antibodies were added to dishes coated with Aβ1-42 protofibrils. The association and dissociation rate constants were obtained by monitoring binding during consecutive incubation steps with two concentrations (300 pM and 1 nM) of the antibodies for 2.5 h and 3 h respectively, followed by a phase where the antibodies were removed to measure dissociation rate. Among the three tested antibodies, Hexa-RmAb158 displayed a much slower dissociation with an almost straight dissociation curve (Fig. 4). Kinetic evaluation was done using a 1:1 curve fit. Here, the association event was detected when the first binding site of an individual antibody binds, while for the appearance of signal reduction for the dissociation phase, all binding interactions of the individual antibody need to be dissociated at the same time and hence the antibody not attached at all. Kinetic evaluation of the binding curve for RmAb158 showed an association rate constant (*k*_a_) of 1.5 × 10^5^ M^−1^s^−1^, and a dissociation rate constant (*k*_d_) of 6.5 × 10^–6^ s^−1^. The affinity, *K*_D_, of the antibody to Aβ protofibrils was determined to be 43 pM. Regarding Tetra-RmAb158 binding to Aβ protofibrils, the antibody displayed an association rate constant of 1.9 × 10^5^ M^−1^s^−1^ and a dissociation rate constant of 4.5 × 10^–6^ s^−1^, with an affinity *K*_D_ of 24 pM (Fig. 4). Kinetic evaluation of Hexa-RmAb158 binding to Aβ protofibrils showed an association rate constant of 2.0 × 10^5^ M^−1^s^−1^. For Hexa-Rmab158, the dissociation rate was very low (almost a straight line, see Fig. 4) and for an accurate estimate of the off-rate and affinity, the fitting was performed in two steps where first the dissociation rate was established followed by fitting the association rate constant while using the dissociation rate from the first step. This resulted in a *k*_d_ value of 2.1 × 10^–7^ s^−1^, and an affinity *K*_D_ of 1 pM (Fig. 4), which were ~ 40 times lower than the *k*_d_ and *K*_D_ values of RmAb158. These results indicate that after incubating the antibodies for several hours with a protofibril-coated layer, additional scFv units lead to a more stable and higher-affinity binding of the antibodies.

**Fig. 5:**
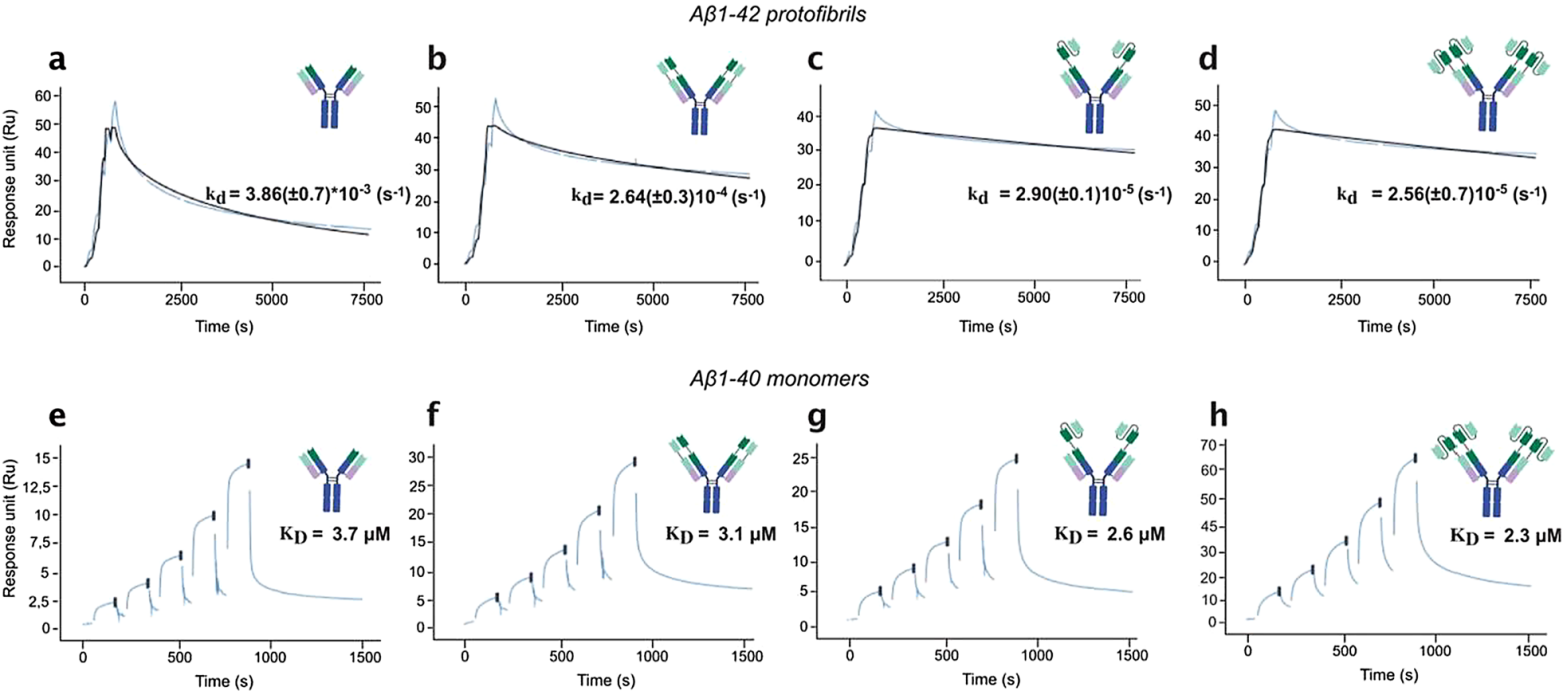
Surface plasmon resonance (SPR) experiments illustrating the binding strength of the recombinant antibodies to Aβ. **a-d** Representative sensorgrams for the recombinant antibodies’ binding to immobilized Aβ1-42 protofibrils. Three-fold dilution series of the recombinant antibodies was used to make single cycle kinetic measurements (1.23, 3.7, 11.1, 33.3 and 100 nM). The dissociation rate constants (*k*_d_) with standard deviations for the four antibodies were calculated using a one-to-one kinetic model. Lower rate of dissociation from protofibrils is measured in Tetra-RmAb158 (**c**) and Hexa-RmAb158 (**d**). **e–h** Representative sensor grams for Aβ1-40 monomers binding to immobilized recombinant antibodies. Two-fold dilution series of Aβ1-40 monomers used (4000, 2000, 1000, 500 and 250 nM). Lines were fitted using steady state kinetic model. The four antibodies exhibited similar steady-state affinity with *K*_D_ values in the micromolar range. Results are generated from two to five repetitive experiments

The binding strength of the recombinant antibodies to Aβ1-42 protofibrils was also determined using SPR (Fig. 5a-d). With SPR, it is more difficult to measure the kinetic properties for as long times as with LigandTracer, which is desired in the case of antibodies with slow rate of dissociation. The curve fit to the dissociation phase was good and there was a large difference in the dissociation rate. RmAb158 demonstrated a *k*_d_ of 3.86 × 10^–3^ s^−1^ (Fig. 5a). DVD-RmAb158 demonstrated an almost 10 times slower dissociation with a *k*_d_ value of around 2.64 × 10^–4^ s^−1^ (Fig. 5b). Binding of Tetra-RmAb158 and Hexa-RmAb158 to Aβ1-42 protofibrils displayed a *k*_d_ value of 2.90 × 10^–5^ s^−1^ and 2.56 × 10^–5^ s^−1^, respectively. These *k*_d_ values were more than 100 times lower than that of RmAb158 (Fig. 5c, d).

The binding strength of the recombinant antibodies to Aβ1-40 monomers was determined with SPR using the steady-state affinity model. The four antibodies displayed weak binding with a *K*_D_ value in the range of 2–4 μM (Fig. 5e-h).

### Evaluation of the binding strength of the multivalent antibodies to different sizes of Aβ aggregates

The second part of our study aimed at comparing the binding of the different antibody formats to different sizes of soluble Aβ aggregates.

### Generation of cross-linked Aβ aggregates of different sizes

**Fig. 6:**
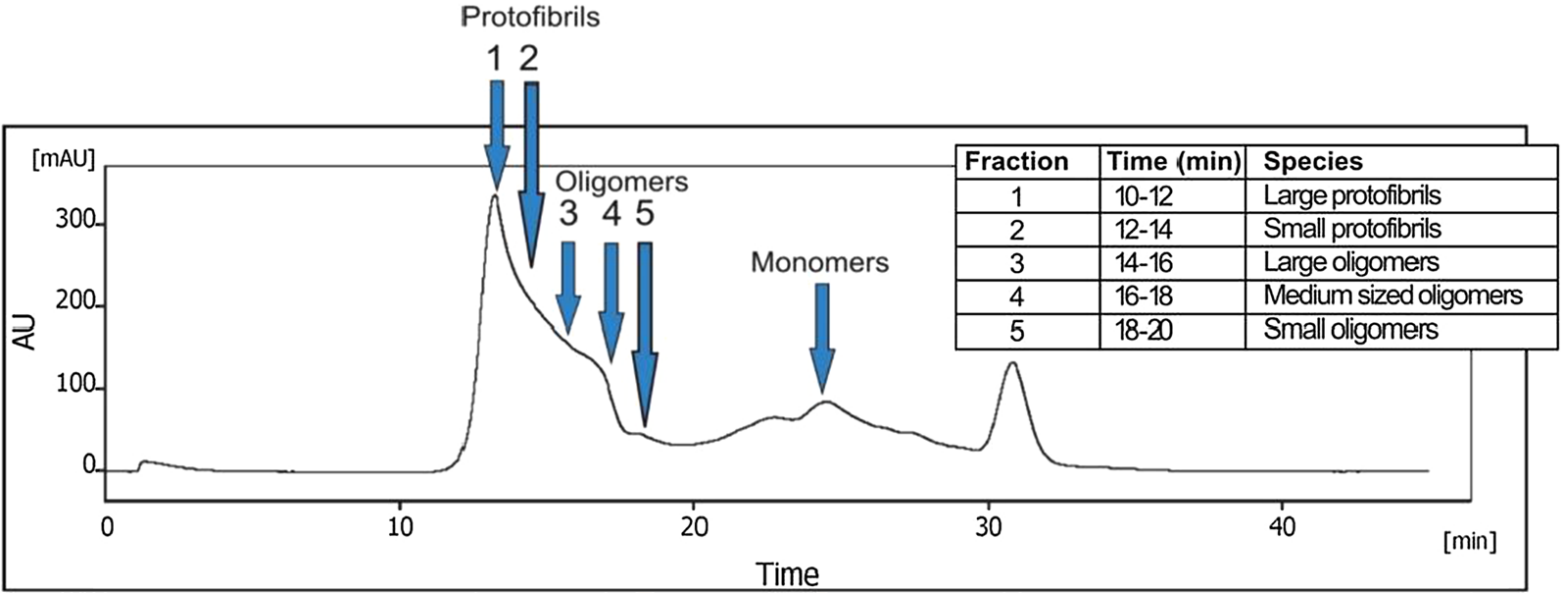
Generation of cross-linked Aβ aggregates of different sizes. Chromatogram showing the different species of the first batch of cross-linked aggregated Aβ1-42 separated by SEC using a Superdex200 column. The peak at ~ 30 min corresponds to retention of salts or degraded amino acids from Aβ

Stable Aβ1-42 aggregates of different sizes were generated using the PICUP method followed by fractionation through SEC using a Superdex 200 column (GE Healthcare). The HPLC-SEC chromatogram confirmed the presence of aggregates of different sizes collected in different fractions, with fraction-1 having the largest size and fraction-5 having the lowest MW (Fig. 6). Synthetic monomers of Aβ had a size of ~ 4.5 kDa. What is defined as Aβ oligomer and protofibril varies among studies. In the current study, Aβ protofibrils were defined as structures with a size of more than 100 kDa that remain soluble after centrifugation at 16,000 × g, and oligomers as soluble aggregates smaller than this. Therefore, fractions 1 and 2 in the SEC chromatogram were defined as large and small protofibrils respectively. Fraction 3 was defined as large oligomers, fraction 4 as medium-sized oligomers and fraction 5 as small oligomers (Fig. 6).

### Evaluation of the binding strength of the recombinant multivalent antibodies to different sizes of cross-linked Aβ1-42 species with ELISA

**Fig. 7:**
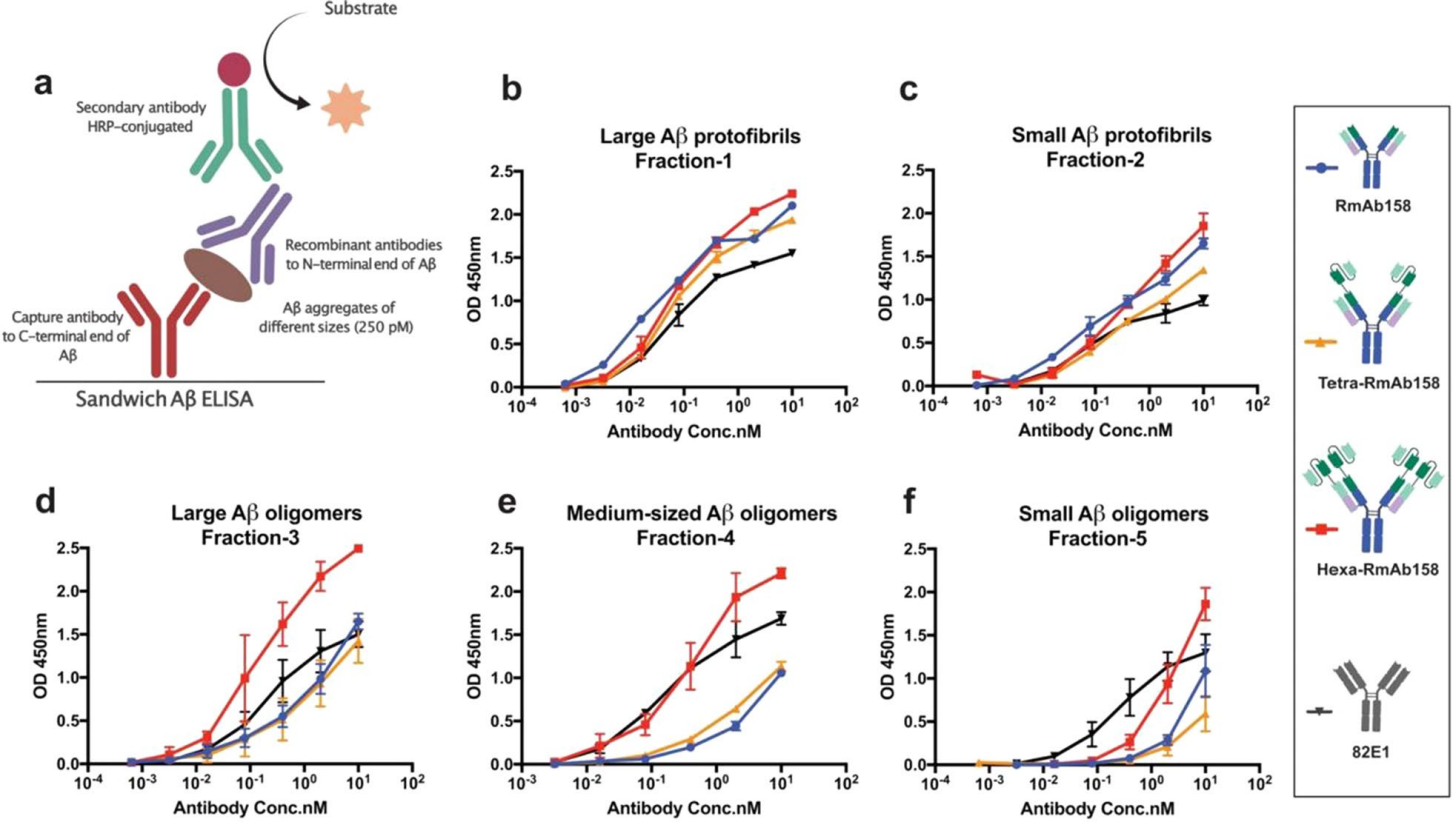
Sandwich ELISA displaying the binding curves of the different antibodies to different species of Aβ. **a** Schematic representation of sandwich Aβ ELISA setup where the antigens (cross-linked Aβ42 fractions) are captured by an antibody coated on the well surface. The capture antibody binds to the C-terminal end of the antigen. This is followed by the addition of the generated recombinant antibodies that detect the N-terminal part of the antigen. **b**, **c** To the large Aβ1-42 aggregates, corresponding to fractions 1 and 2, similar binding strengths were seen among all antibodies. **d**, **e** Hexa-RmAb158 displayed stronger binding than all the other antibodies to the medium-sized aggregates corresponding to fractions 3 and 4. **f** Hexa-RmAb158 displayed some binding to the low-sized aggregates corresponding to fraction 5. The antibody 82E1 has been reported to bind equally strong to both monomers and aggregates and was used as a control. Data are presented as mean ± SD, *n* = 2 for fractions 1 & 4, *n* = 4 for fractions 2, 3 & 5

Using a sandwich ELISA sparsely coated with an antibody that binds the C-terminal end of Aβ, we evaluated the binding strength of the multivalent antibodies to Aβ aggregates that vary in size. We used this type of sandwich ELISA with two-hour-long incubations to detect binding to differently sized cross-linked Aβ1-42 aggregates (Fig. 7a). Results showed that all antibody formats showed a similar response to the large cross-linked aggregates (protofibrils, fractions 1 and 2, > 100 kDa), regardless of the valency (Fig. 7b, c). The method was not sensitive enough to detect differences between very strong binders. However, antibody binding to the smaller aggregates was expected to be substantially reduced and hence differences should be detectable. With such sandwich ELISA setup, we also evaluated the binding strength of the different antibodies to the cross-linked Aβ1-42 fractions 3 and 4 containing the medium-to-large oligomers of around 60–100 kDa. Results showed that the Hexa-RmAb158 bound stronger than the other recombinant antibodies to fractions 3 and 4 (Fig. 7d, e). The Hexa-RmAb158 also displayed some binding to the small cross-linked oligomers at nanomolar concentrations, while the other recombinant antibodies displayed weaker binding (Fig. 7f). The antibody 82E1 has been reported to bind to Aβ of different sizes with similar binding strengths [46, 47] and was used as a control here. The DVD-RmAb158 antibody showed a very weak binding strength to almost all the cross-linked Aβ1-42 fractions in this experiment. This antibody format was more prone to aggregate and required very sensitive handling and was therefore not included in all our experiments. The binding strength of each individual antibody to the different fractions of Aβ from the same experiment is illustrated in Additional file 1: Fig. S2.

To estimate the size of Aβ oligomers that can best generate an avidity effect by binding Hexa-RmAb158, we generated another batch of cross-linked Aβ1-42 oligomers (Additional file 1: Fig. S3a-b) where we could collect fractions containing even smaller oligomers than those generated before (Fig. 6). In the same sandwich ELISA setup as described previously, Hexa-RmAb158 displayed the best binding strength to aggregates with a size range of 50–200 kDa (Additional file 1: Fig. S3c). Nonetheless, the avidity of Hexa-RmAb158 to Aβ1-42 decreased in fractions with size < 50 kDa (Additional file 1: Fig. S3d–f). These results indicate that oligomers of ~ 50 kDa and above are the species of Aβ that can be best detected with Hexa-RmAb158. As expected from the previous results, the other tested recombinant antibodies also bound weaker to small aggregates in the concentration range used (Additional file 1: Fig. S3d–f).

**Fig. 8:**
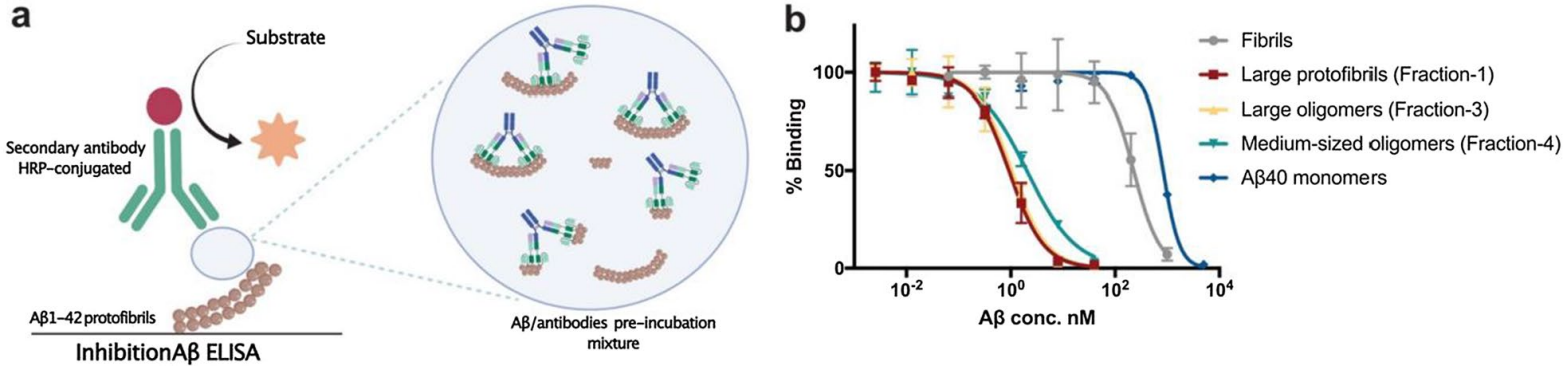
Inhibition ELISA illustrating the different binding strengths of Hexa-RmAb158 to Aβ. Five different species of Aβ used in this setup: insoluble fibrils, protofibrils in fraction 1, oligomers in fraction 3, oligomers in fraction 4, and Aβ40 monomers. **a** Schematic representation of inhibition Aβ ELISA, where a pre-incubated Aβ/antibody mixture is added to a protofibril-coated plate. **b** Hexa-RmAb158 displaying a highly selective binding to the protofibrils and both the oligomeric fractions, with moderate binding strength to the fibrils and weak binding to the monomers. The concentrations were log transformed, and the obtained OD values were normalized to 100% binding, where the highest OD value is defined as 100% binding, and OD value of zero is defined as 0% binding. Data are presented as mean ± SD, *n* = 2–3

To investigate if Hexa-RmAb158 bound stronger to oligomers and protofibrils  than to insoluble fibrils and monomers, an inhibition ELISA was performed. In this setup, a pre-incubated Aβ aggregate and Hexa-RmAb158 mixture was added to a protofibril-coated ELISA plate (Fig. 8a). The same setup has previously been used to display strong binding of mAb158 to Aβ protofibrils, while having intermediate and weak bindings to fibrils and monomers respectively [31, 35, 36]. In this ELISA setup, the ability of the different preincubated Aβ aggregates to inhibit Hexa-RmAb158 binding to the protofibrils bound to the plate should be related to the antibodies’ binding strength. The Aβ species used in this ELISA setup were sonicated insoluble Aβ1-42 fibrils prepared as described previously [31], cross-linked Aβ1-42 protofibrils (fraction 1 in the SEC chromatogram, Fig. 6), two smaller sizes of cross-linked Aβ1-42 oligomers (fractions 3 and 4 in the SEC chromatogram, Fig. 6) and Aβ1-40 monomers. Because Aβ1-42 is more prone to aggregate, Aβ1-40 was used to assess monomer binding as it is more stable as a soluble monomer [48]. Hexa-Rmab158 displayed a similar strong binding to the three fractions of cross-linked Aβ1-42 (IC50 of 0.9, 1.0 and 2.0 nM, respectively) (Fig. 8b). Importantly, Hexa-RmAb158 exhibited moderate binding strength to sonicated Aβ1-42 fibrils (IC50 227 nM), and weak binding to Aβ1-40 monomers (IC50 834 nM) (Fig. 8b).

### Hexa-RmAb158 and related antibodies demonstrate no binding to protein aggregates other than Aβ

**Fig. 9:**
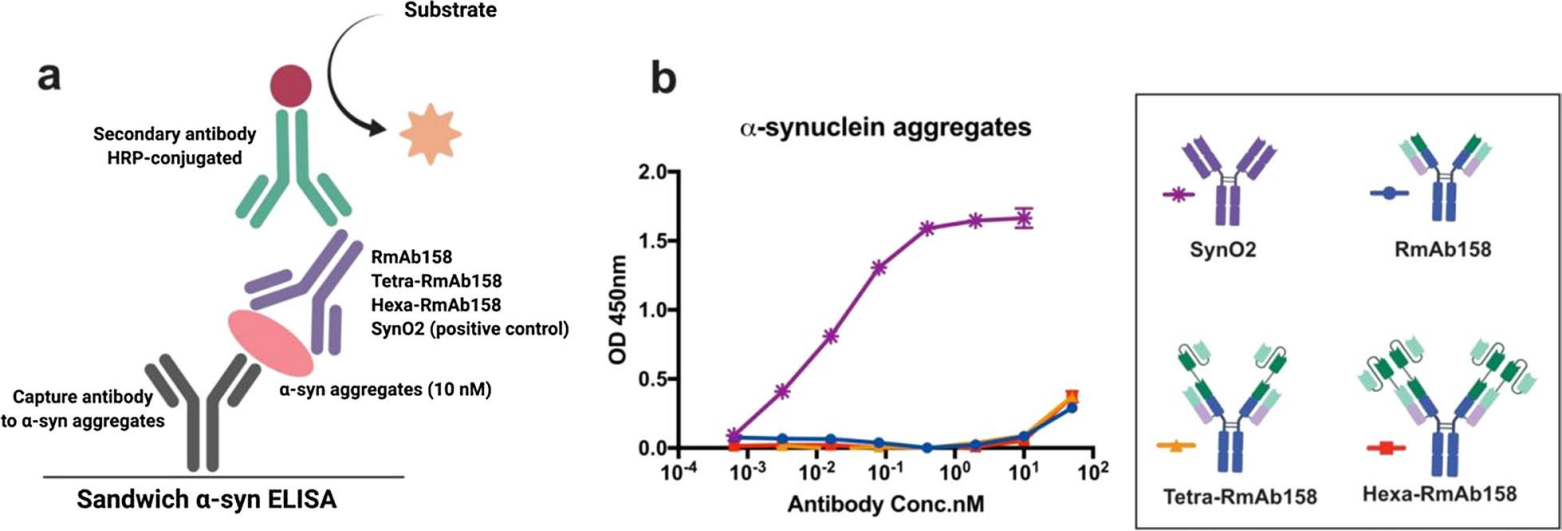
Sandwich ELISA displaying the binding curves of the different antibodies to α-synuclein aggregates. **a** Schematic representation of sandwich ELISA setup where the antigen (α-synuclein)  was captured by an antibody coated on the well surface. This is followed by the addition of the generated recombinant antibodies. **b** Hexa-RmAb158, Tetra-RmAb158 and RmAb158 displayed no binding to α-synuclein aggregates. The control antibody SynO2 displayed a strong binding to α-synuclein aggregates. Data are presented as mean ± SD, *n* = 3

To display that Hexa-RmAb158 does not recognize aggregates of other amyloidogenic proteins, a sandwich ELISA was applied (Fig. 9a) detecting α-synuclein oligomers, the pathological aggregates in Parkinson’s disease. The anti α-synuclein antibody (SynO2) was used as a positive control [41]. Hexa-RmAb158, Tetra-RmAb158 and RmAb158 showed no binding to α-synuclein aggregates (Fig. 9b).

### The hexavalent antibody protects neuronal cells from the toxic effects of Aβ aggregates

**Fig. 10:**
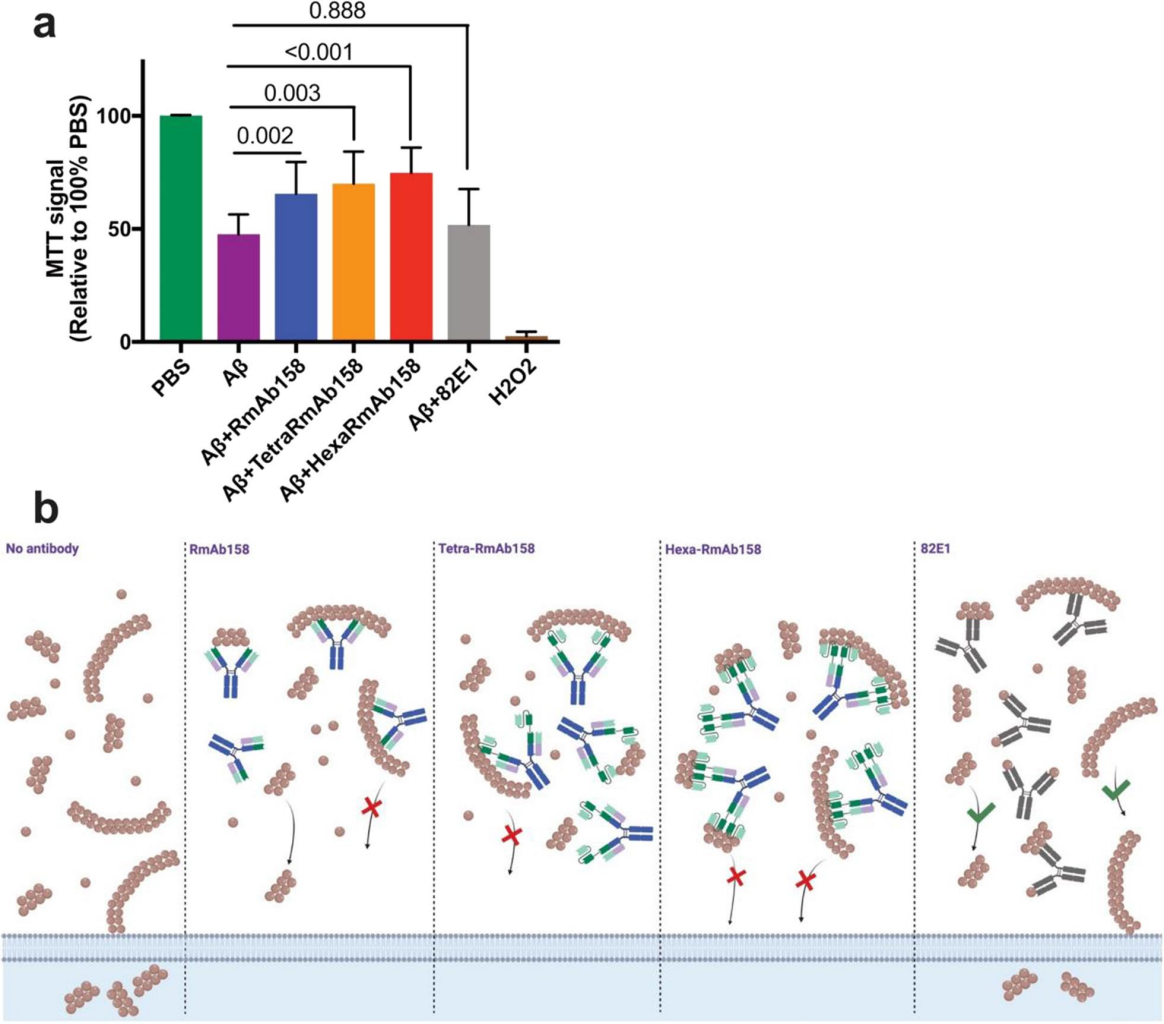
Metabolic activity of neuro2a cells after treatment with Aβ and recombinant antibodies measured by MTT assay. **a** Cells were incubated for 24 h with Aβ1-42 aggregates alone (500 nM) or in combination with 150 nM of the antibodies. PBS was used as a negative control and H_2_O_2_ as a positive control in all the experiments. Results are presented as MTT signal relative to PBS as 100%. Results are obtained from two repetitive experiments, where five to six replicates were used in each experiment. Data are presented as mean ± SD. One-way ANOVA was used followed by Dunnett’s post hoc test. A *P*-value of less than 0.05 refers to the presence of statistically significant differences. **b** Schematic representation of the ability of RmAb158, Tetra-RmAb158 and Hexa-RmAb158 to reduce cell metabolism impairments induced by a mixture of Aβ

The efficacy of the different antibodies in vitor was studied by investigating their ability to protect mouse neuroblastoma Neuro2a cells from Aβ-induced effects on cell metabolism using MTT assay. Since the concentration of the separated fractions of cross-linked Aβ42 aggregates (Fig. 6) was too low to induce reduction in MTT signal, we performed this experiment with a heterogenous mixture of cross-linked Aβ1-42 aggregates, which also included some proportion of monomers. After over 24 h of incubation, 500 nM of Aβ1-42 mixture resulted in a reduction of MTT signal by ~ 53% (MTT signal 47% relative to 100% of PBS-treated cells) (Fig. 10a). The reduced MTT signal was significantly reversed to ~ 65% by RmAb158 (*P* = 0.002), to ~ 70% by Tetra-RmAb158 (*P* = 0.003), and to 74% by Hexa-RmAb158 (*P* < 0.001) (Fig. 10a). The control antibody 82E1 that has a high affinity to the non-toxic monomers as well as the aggregates did not significantly reduce the Aβ1-42 induced toxic effects (Fig. 10a).

## Discussion

Passive immunotherapies using monoclonal antibodies are in ongoing clinical trials for the treatment of AD. Some of these trials have been discontinued due to the lack of efficacy in slowing cognitive impairment [49–51]. A high incidence of adverse events such as amyloid-related imaging abnormalities with edema (ARIA-E), possibly caused by binding to fibrils in the vessel walls, have been seen in some of the trials [52, 53].

The aim of the current study was to enhance the avidity of monoclonal antibodies through the design of multivalent antibody formats and investigate their binding strength to soluble aggregated species of Aβ. These properties are desired because of the high cytotoxicity of the intermediate soluble species of Aβ [10, 12, 54]. In addition, increasing the avidity will also decrease the rate of dissociation from the soluble Aβ aggregates.

To this end, we have designed, produced and evaluated binding characteristics of multivalent antibodies to different aggregated Aβ species, but also to physiologically abundant monomers. The designs were based on RmAb158, an antibody that has previously been shown to have high binding strength to protofibrils due to avidity [31, 34]. The humanized version of RmAb158, lecanemab (BAN2401), is currently in a phase 3 clinical trial with 1795 participating patients with early and prodromal AD. In a phase 2b trial including 856 patients with AD, lecanemab (BAN2401) demonstrated a remarkable biological activity in lowering Aβ levels in the brain with a good safety profile [55].

We utilized the SPR and LigandTracer techniques to compare the binding of the antibody constructs to protofibrils, since these technologies can establish the affinity and avidity without the necessity of reaching equilibrium in the binding process as with ELISA. For a high avidity binding, as displayed by Hexa-RmAb158 to Aβ protofibrils, 95% of the equilibrium binding will only, theoretically, be reached after incubation for roughly 17 days when having a concentration similar to the apparent affinity for the interaction. Required incubation times are even longer for lower concentrations. The obtained results from both methods revealed a lower rate of dissociation with Hexa-RmAb158, which showed a slower dissociation from Aβ protofibrils when compared to RmAb158 (Figs. 4, 5). This can be explained by the additional binding domains of Hexa-RmAb158 that provide a higher number of antigen–antibody interaction sites. If one of the paratopes dissociates from its interaction site, there are additional paratopes that are still associated with the target in the case of Hexa-RmAb158. In comparison, Tetra-Rmab158 will have fewer available binding sites, and RmAb158 has only one binding site available. Therefore, a complete dissociation of the hexavalent antibody from its target requires very long times. The dissociation rates measured in the SPR are faster than those from the LigandTracer, which could be due to the longer incubation periods enabling more time for multivalent binding and how the protofibrils are connected to the surface. In LigandTracer, unmodified protofibrils are bound directly to the plate, while in the SPR, the protofibrils are bound to the sensorchip with amine coupling. Since the N-terminal end of Aβ1-42 is the epitope  for mAb158 [31], it is likely that this is the cause for the weaker binding detected in the SPR, but the relative difference in avidity should still be rather correct. By comparing the measured dissociation process and the one estimated by the 1:1 fit in SPR, it can be seen that the actual dissociation is more heterogeneous with a fraction that dissociates more rapidly, leaving more stably bound fraction behind that dissociates more slowly. This heterogeneity might be related to a variation in multivalent binding on the SPR sensorchip, where some antibodies do not manage to bind with all possible binding sites. The advantage of real-time interaction analysis with LigandTracer over SPR is that it allows monitoring of binding to coated layer of Aβ protofibrils while having longer incubation times to allow reaching of a binding equilibrium.

One of the complications in designing Aβ oligomer antibodies is the cross-reactivity with insoluble fibrils and monomers. Several antibodies have been designed to target Aβ oligomers, but they have been shown to also bind to fibrillar and monomeric species. Strong binding of antibodies to Aβ fibrils is likely to be associated with an increased risk of ARIA-E, which is a frequently observed adverse event in AD clinical trials with monoclonal antibodies [56]. In addition, the monomeric Aβ might play a physiological role and exist in higher amounts than oligomers [15, 57]. Binding to Aβ monomers in the periphery could also interfere with the ability of the antibodies to reach their intra-brain target. Therefore, antibodies that selectively bind to the oligomers and protofibrils, with low binding to fibrils and monomers, are desired. Here, we showed with inhibition ELISA that the Hexa-RmAb158 has a much stronger binding to the oligomers and protofibrils, which is ~ 200 times stronger than its binding to the insoluble fibrils, and 800 times stronger than its binding to the monomers. This has previously been demonstrated with mAb158 as well using similar inhibition ELISA setup [31, 35]. Furthermore, our ELISA experiments displayed no binding of Hexa-RmAb158 and related antibodies to α-synuclein aggregates, further confirming the previous report of no binding of the parental mAb158 antibody to protein aggregates other than Aβ [31]. This suggests that binding of Hexa-RmAb158 is specific to the N-terminal of Aβ and not directed towards a structural element common for different amyloidogenic protein aggregates. Being able to strongly bind the soluble oligomeric aggregates of Aβ could be of therapeutic benefit, since it has been shown that the soluble Aβ aggregates are toxic to neurons and associated with enhanced release of inflammatory cytokines [58, 59]. This can be explained by their colocalization with several pre- and post-synaptic markers, faster induction of apoptotic changes and activation of the mitochondrial death pathway. In addition, previous research has shown that the small soluble Aβ1-42 aggregates have a higher permeability to cell membranes [21]. In our study, Hexa-RmAb158 was efficient in reducing cell metabolism impairment caused by Aβ of different sizes (Fig. 10a), which could be attributed to the selectivity and low dissociation rates of this antibody to the soluble toxic aggregates of Aβ.

Our approach  to designing multivalent antibodies could also be applied for other diseases caused by aggregated proteins or repetitive targets. Multivalent antibody designs could provide higher chances for their paratopes to rebind to their targets with decreased dissociation rates, which in turn, could aid in keeping of antibodies in areas around the target. Strong and continuous occupancy of targets could be of particular importance in cancer immunotherapy, where studies have displayed heterogenous and non-tumor specific distribution of antibodies [1]. It is however worth mentioning that enhancing the avidity of antibodies is also dependent on the nature and the surface size of the targeted antigen.

## Conclusion

In conclusion, we have developed a hexavalent antibody that can bind with high avidity to soluble aggregates of Aβ including small oligomers, while having low binding to the physiological monomers. Due to the decrease in the rate of dissociation, the binding strength of the hexavalent antibody to protofibrils is enhanced by 40 times when measured using real-time interaction analysis with LigandTracer. The binding of the antibody to different Aβ species and the ability to reduce cell death from the toxic effects of Aβ illustrate the potential of such multivalent designs for the generation of diagnostic and therapeutic interventions in the future. Reformatting antibodies to customize the binding profile to targets is possible for many antibodies with extensive use beyond AD.

## Supplementary Information

**Additional file 1.Fig. S1.** IgG antibody and 12-mer Aβ oligomer binding.** Fig. S2.** Sandwich ELISA displaying the binding properties of each antibody to different cross-linked fractions of Aβ 1-42 generated in Fig. 6.** Fig. S3.** Sandwich ELISA displaying the binding curves of the different antibodies to another batch of cross-linked Aβ 1-42 fractions.

## Data Availability

The datasets used and/or analysed during the current study are available from the corresponding author on reasonable request.

## Abbreviations

Aβ: Amyloid-β
AD: Alzheimer’s disease
ARIA-E: Amyloid-related imaging abnormalities with edema
DVD: Dual variable domain
PICUP: Photo-induced cross-linking method
scFv: Single chain fragment variable
SPR: Surface plasmon resonance
