# Supplementary material for: Novel multivalent design of a monoclonal antibody improves binding strength to soluble aggregates of amyloid beta
Source: Transl Neurodegener. 2021 Sep 28;10:38. doi: 10.1186/s40035-021-00258-x (PMC8477473; doi:10.1186/s40035-021-00258-x)
Supplement: Supplementary file 1 — Additional file 1.Fig. S1. IgG antibody and 12-mer Aβ oligomer binding. Fig. S2. Sandwich ELISA displaying the binding properties of each antibody to different cross-linked fractions of Aβ 1-42 generated in Fig. 6. Fig. S3. Sandwich ELISA displaying the binding curves of the different antibodies to another batch of cross-linked Aβ 1-42 fractions. [file 40035_2021_258_MOESM1_ESM.docx]

**Supplementary Information**

**
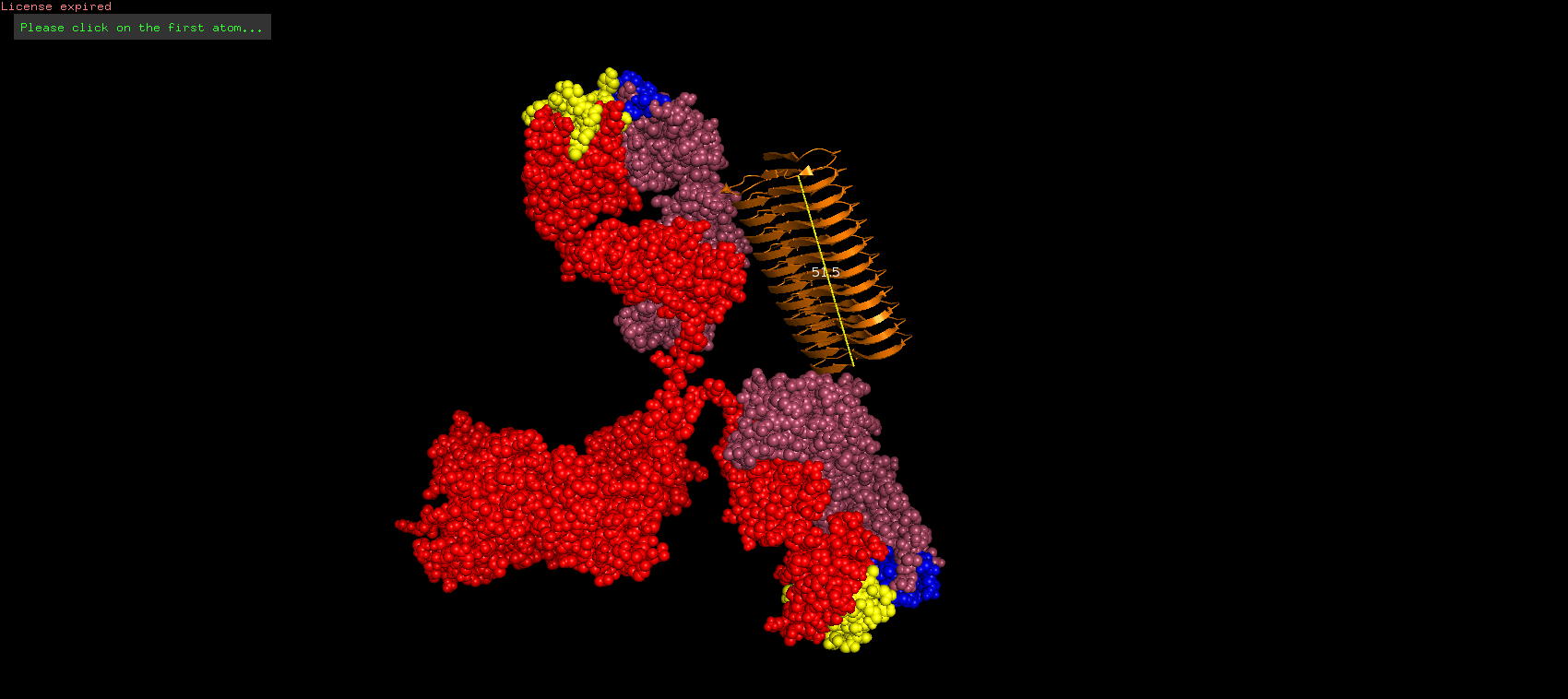
**

**Fig. S1 IgG antibody and 12-mer Aβ oligomer binding**. Overlay montage showing the crystal structure of a reference IgG4 antibody (PDB ID 5DK3) in the same picture as a 12-mer Aβ oligomer (PDB ID 2BEG). The spatial distance between the two CDR regions of an IgG4 antibody (yellow and blue) in this structure is substantially larger than that of an oligomer consisting of 12 monomers (orange) that is 51.5 nm long. This is a snapshot of the crystal structure of the antibody and the arms can be flexible. However, it has previously been shown with NMR that the spatial distance between the two paratopes of IgG antibodies is around 100 Å (ref 32).

Heavy chain (red), light chain (raspberry), CDR-heavy chain (yellow), CDR-light chain (blue). Figure created using PyMol.

**
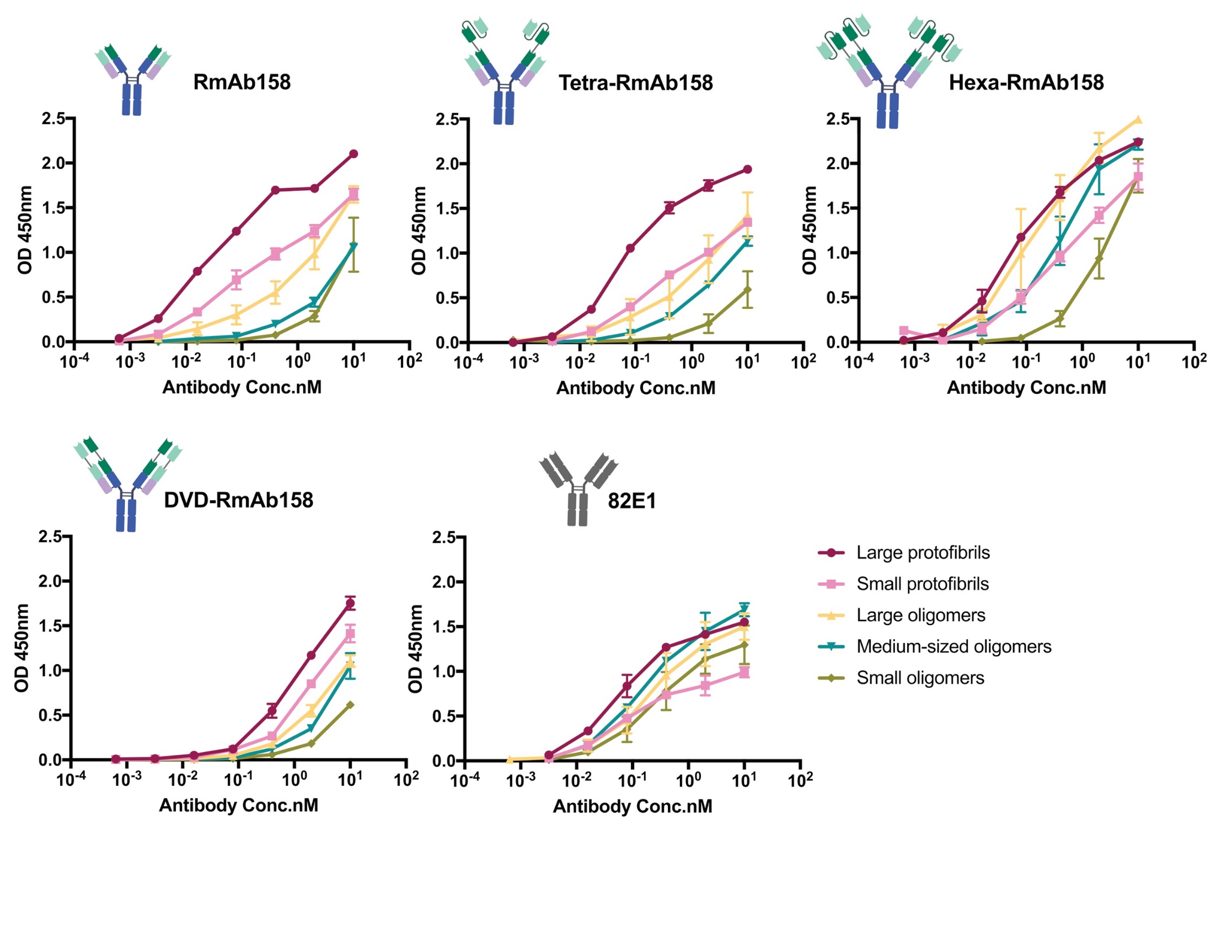
**

**Fig. S2** **Sandwich ELISA displaying the binding properties of each antibody to different cross-linked fractions of Aβ1-42 generated in Fig. 6**. Data are presented as mean ± SD, *n* = 2 for large protofibrils & medium-sized oligomers, *n* = 4 for small protofibrils, large oligomers and small oligomers.

**
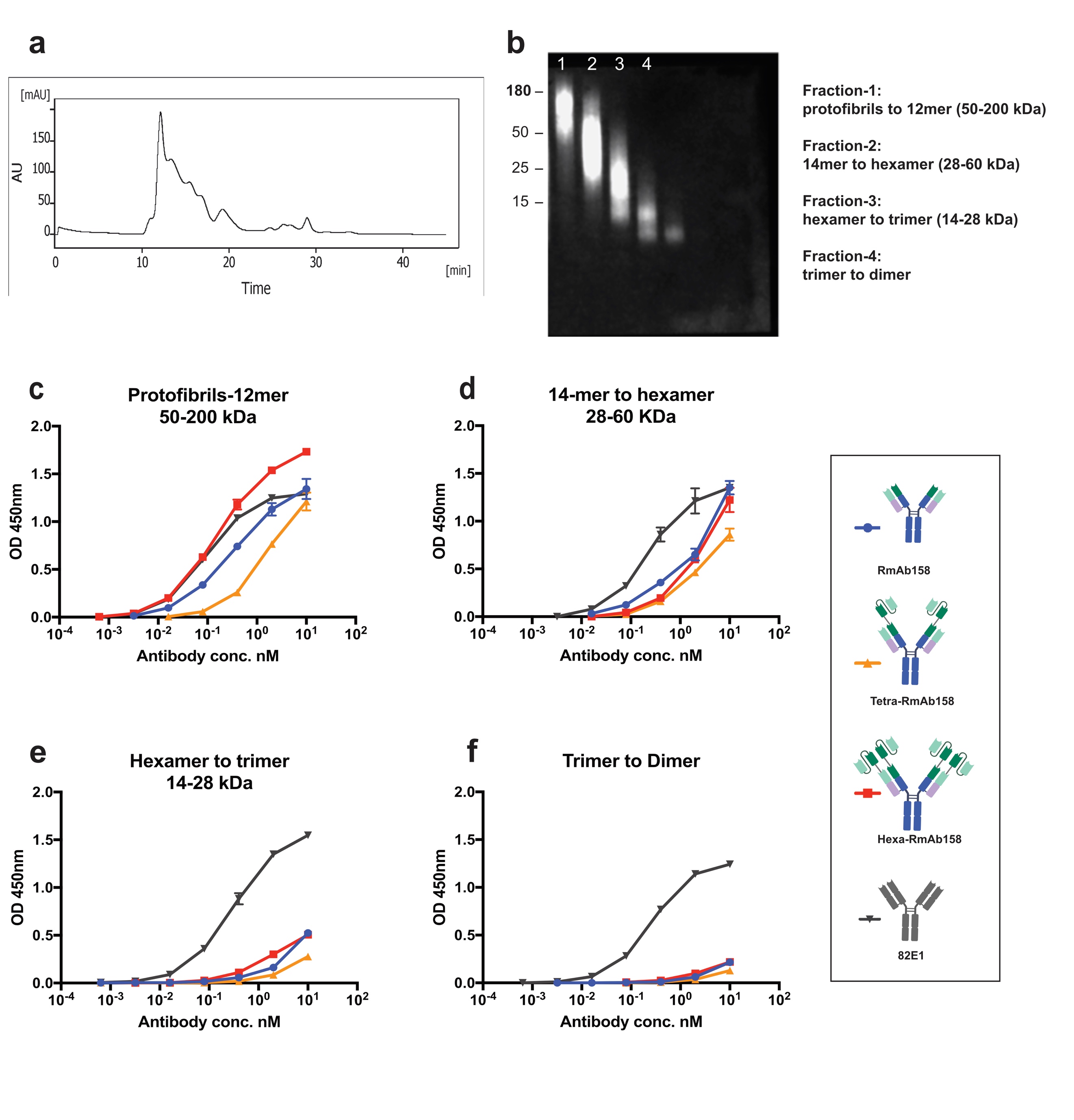
**

**Fig. S3** **Sandwich ELISA displaying the binding curves of the different antibodies to another batch of cross-linked Aβ1-42 fractions**. (**a**). Chromatogram showing the different species of the second batch of cross-linked aggregated Aβ1-42 separated by SEC using a Superdex 75 column. (**b**). SDS-PAGE western blot analysis showing the estimated size of the new batch of cross-linked Aβ1-42 fractions. (**c**). Hexa-RmAb158 displayed stronger binding to aggregates in the size range of 50-200 kDa compared to the reference antibody. (**d**). Hexa-RmAb158, RmAb158 and Tetra-RmAb158 displayed weaker binding to aggregates in the size range of 28-60 kDa compared to aggregates of 50-200 kDa. (**e** and **f**) No binding to Aβ1-42 trimers and dimers in the concentration range used (10 nM- 3.2 pM). The control antibody 82E1 bound equally well to all fractions. Data are presented as mean ± SD, *n* = 2.
